# How to Breathe

**Published:** 1890-02

**Authors:** 


HOW TO BREATHE.—The following heretofore unheard of information in regard to the breath and breathing was made public in Kentucky recently by a school-boy of twelve years, who wrote an essay on the subject:

"We breathe with our lungs, our lights, our kidneys and our livers. If it wasn't for our breath we would die when we slept.

Our breath keeps the life a-going through our nose when we are asleep. Boys who stay in a room all day should not breathe. They should wait until they get out in the fresh air. Boys in a room make bad air called carbonicide. Carbonicide is as poison as mad dogs. A lot of soldiers were once in a black hole in Calcutta, and carbonicide got in there and killed them. Girls sometimes ruin the breath with corsets that squeeze the diagram. A big diagram is the best for the right kind of breathing."—*Medical Classics.*

